# A new era of thromboelastometry

**DOI:** 10.1590/S1679-45082017MD3130

**Published:** 2017

**Authors:** Tomaz Crochemore, Felipe Maia de Toledo Piza, Roseny dos Reis Rodrigues, João Carlos de Campos Guerra, Leonardo José Rolim Ferraz, Thiago Domingos Corrêa

**Affiliations:** 1 Hospital Israelita Albert Einstein, São Paulo, SP, Brazil.

**Keywords:** Hemorrhage/prevention & control, Blood transfusion/adverse effects, Goal-directed therapy, Thrombelastography/methods, Hemostatic drugs, Rotational thromboelastometry, Hemorragia/prevenção & controle, Transfusão de sangue/efeitos adversos, Terapia guiada por metas, Tromboelastografia /métodos, Drogas hemostáticas, Tromboelastometria rotacional

## Abstract

Severe hemorrhage with necessity of allogeneic blood transfusion is common complication in intensive care unit and is associated with increased morbidity and mortality. Prompt recognition and treatment of bleeding causes becomes essential for the effective control of hemorrhage, rationalizing the use of allogeneic blood components, and in this way, preventing an occurrence of their potential adverse effects. Conventional coagulation tests such as prothrombin time and activated partial thromboplastin time present limitations in predicting bleeding and guiding transfusion therapy in critically ill patients. Viscoelastic tests such as thromboelastography and rotational thromboelastometry allow rapid detection of coagulopathy and goal-directed therapy with specific hemostatic drugs. The new era of thromboelastometry relies on its efficacy, practicality, reproducibility and cost-effectiveness to establish itself as the main diagnostic tool and transfusion guide in patients with severe active bleeding.

## RESUMO

A hemorragia grave com necessidade de transfusão de sangue e componentes é uma complicação frequente na unidade de terapia intensiva e está associada ao aumento da morbidade e da mortalidade. A identificação adequada e o tratamento precoce da causa específica da coagulopatia tornam-se fundamentais para o controle efetivo da hemorragia, racionalizando a utilização de sangue e componentes, e desta forma, prevenindo a ocorrência de efeitos adversos. Testes convencionais da coagulação (tempo de ativação de protrombina e tempo de tromboplastina parcial ativada) apresentam limitações para prever sangramento e guiar a terapia transfusional em pacientes graves. Testes viscoelásticos como a tromboelastografia e tromboelastometria rotacional permitem a rápida detecção da coagulopatia e orientam a terapia de forma individualizada, alvo dirigida com drogas hemostáticas específicas. A nova era da tromboelastometria confia na sua eficácia, praticidade, reprodutibilidade e custo-eficácia para se firmar como a principal ferramenta diagnóstica e guia transfusional em pacientes com sangramento ativo grave.

## INTRODUCTION

Countless diseases are found in the intensive care setting or operating room that can compromise the coagulation system. Severe hemorrhage with necessity of allogeneic blood transfusion is a frequent clinical manifestation that can lead to undesirable outcomes.^(1)^ Traditionally, allogeneic blood transfusion practice has been indicated based on conventional coagulation tests in association with clinical signs of active bleeding.^(2,3)^Coagulopathy defined as impairment in platelet counts, in coagulation tests and in fibrinogen concentration, is common in critically ill patients increasing the risk of allogeneic blood transfusion. Fresh frozen plasma is associated with a three-fold increase the risk of nosocomial infection in severe surgical patients.^(4)^Platelets concentrate, red blood cells and fresh frozen plasma transfusions are associated with a higher risk of acute pulmonary insufficiency.^(5)^

The hemostatic system is composed by the endothelium, soluble blood proteins, platelets, fibrinolytic and anti-fibrinolytic systems, that are responsible for activation, modulation, and lysis of clot. The cell-based model of coagulation described in 2001 by Hoffman et al., demonstrates that the complex process of clot formation is triggered by the tissue factor and involves four consecutives different phases: initiation, amplification, propagation and clot stabilization.^(6)^

The coagulation system in the critical setting has been assessed traditionally throughout conventional coagulation tests such as prothrombin time, international normalized ratio (INR), thrombin time and activated partial thromboplastin time.^(3)^ Yet, conventional coagulation tests were validated to monitor vitamin K antagonists and heparin therapy. Even tough, conventional coagulation tests have not been validated to predict and/or to guide therapy in acute (acquired) hemorrhage, they have been widely used for this purpose.^(7)^

The absence of thrombomodulin, expressed by endothelial cells, which is responsible for activation of protein C pathway, a natural coagulation inhibitor, limits the evaluation of conventional coagulation tests. conventional coagulation tests only evaluate the thrombin generation determined by pro-coagulation factors, so they are not able of demonstrating the hemostatic balance between coagulation activating and inhibiting factors.^(8,9)^

Conventional coagulation tests reflect poorly the *in vivo* hemostasis since they change when coagulation factors show a *deficit* of more than 50%. Conventional coagulation tests are performed in the plasma sample, thus not take into consideration the interaction of the coagulation factors with platelets, blood cell elements and the vascular endothelium.^(7)^ The influence of hypothermia is not measured, since these tests are performed at 37°C. In this way, complex and multifactoral hemostatic disorders, such as those seen in hemorrhage due to trauma, postpartum, liver diseases, postoperatively and dengue, are difficult to analyze with conventional coagulation tests. Viscoelastic tests have become fundamental for the diagnosis and management of patients with severe hemorrhagic disease. Rotational thromboelastometry (ROTEM^®^) address these gaps providing promptly results, proper inform the dynamics of formation, stabilization and dissolution of clot, reflecting the *in vivo* hemostasis at the bedside.^(10,11)^

Thromboelastography (TEG^®^), described by Hartert in 1948, allows global evaluation of clot formation process, including initiation, formation, stabilization, and lysis of the clot.^(12)^Thromboelastography is a laboratory method that demonstrates the interactions among the blood cells and their biochemical characteristics by means of a graphic representation. Thromboelastography (TEG^®^; Haemoscope Corporation, IL, USA) or thromboelastometry (ROTEM^®^, TEM International GmbH, Munich, Germany) allow a rapid and robust assessment of the clot, using a minute amount of whole blood.^(2)^

The first descriptions of applicability of TEG^®^ were in liver transplants.^(13)^ Later, it was described in cardiac surgery.^(14)^In trauma patients, thromboelastography demonstrated the capacity to anticipate the need for transfusion.^(15)^ In the 1990s, the method went through improvements, the device became more resistant to vibrations, allowing its dislocation to the bedside. The ROTEM^®^ system has a computer for automated analysis, an electronic pipette, four channels for simultaneous measurements and the modern software became the graph more attractive. The use of new reagents as inhibitors and activators accelerated the test results and allowed the identification of different coagulation disorders. In this way, thromboelastometry may guide the hemostatic therapy through goals, according to the need of each patient.^(2)^

Massive hemorrhage and blood transfusions are associated with increased morbidity, mortality, and costs.^(16-19)^ Viscoelastic tests (TEG^®^ and ROTEM^®^) can reduce blood transfusion needed and may optimize the treatment of severely ill patients, since they guide and individualize treatment, justifying investment in this cost-effective technology.^(20,21)^The new era of thromboelastometry relies on its efficacy, practicality, reproducibility and cost-effectiveness to establish itself as the main diagnostic tool and transfusion guide in patients with severe active bleeding.

## METHODOLOGY OF THE VISCOELASTIC TESTS

For the performance of the viscoelastic tests (ROTEM^®^ or TEG), one citrated blood sample is needed, collected by venous puncture of peripheral blood. This may be done at the patient’s temperature, which represents an advantage to patients with blood dyscrasia related to hypothermia.

**Figure 1:**
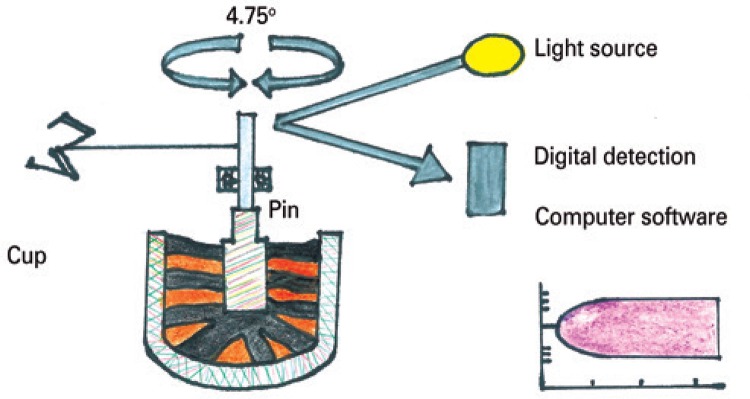
Schematic representation of analysis with rotational thromboelastometry

The principle of TEG^®^ involves the incubation of 360uL of whole blood at 37°C, in a heated cylindrical cup. The cup oscillates during 10 seconds at an angle of 4°45’ in a bowl with a pin freely suspended by a twisted wire (Figure 1).

**Figure 2:**
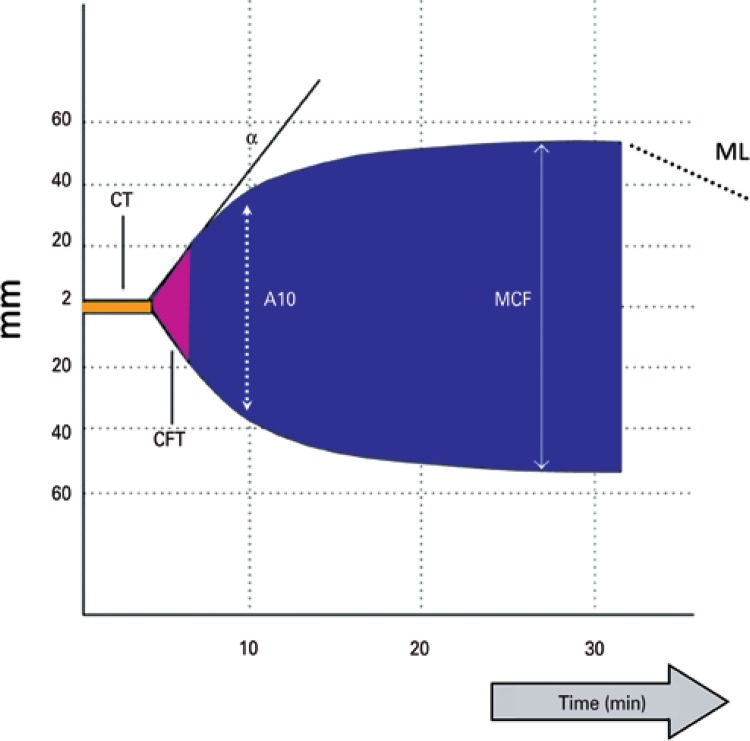
Rotational thromboelastometry parameters

CT: clotting time; CFT: clot formation time; MCF: maximum clot firmness; ML: maximum lysis; A10: amplitude 10 minutes.

The biochemical changes occur in pH, electrolytes, and temperature, promoting interaction among blood cells and the subsequent bond between fibrins and platelets, which by the cup’s rotating movement, transmits a movement pace to the immersed pin. In this way, the magnitude of the graphic representation is directly related to the resistance of the clot formed. After retraction of the clot, its lysis occurs. The bonds are broken, and the transfer of movement of the cup is reduced. The movement of the pin rotation through the mechanical transducer is an electric signal, manifested graphically (Figure 2).

With the ROTEM^®^, contrary to the TEG^®^, it is the steel pin that makes a 4°75’ rotation relative to the cup. By optic reading, this movement transmits to software a graphic representation of amplitude relative to the time of the entire process of clot formation, from its initiation, maximal formation up to its lysis (Figure 1). The advantage the ROTEM^®^ offers is its capacity to present results within 5 to 30 minutes, due to accelerating and inhibiting reagents of the coagulation process.^(10)^

The resulting hemostasis profile is a measure of the time it takes for the first chains of fibrin to be formed, the kinetics of clot formation, the resistance of the clot, and finally, of its dissolution. Physical properties of the clot depend on the relation among fibrinogen, platelets, and plasma proteins. This process produces a characteristic graphic tracing that reflects the different phases of coagulation, allowing its qualitative evaluation (Figure 2).^(22)^

## VARIABLES AND CHANNELS FOR THROMBOELASTOMETRY ANALYSIS

Rotational thromboelastometry is the graphic representation of viscoelastic changes during whole process of fibrin polymerization. From formation to lysis, its variables are time, dynamics, size, and firmness of the clot (Figure 2):

- Clotting time (CT), from zero 0 to 2mm: it corresponds to the beginning of the analysis until the start of clot formation. It is the onset of thromboplastin activation, with the formation of the first fibrins, which reach a 2mm amplitude. This is the beginning of the clot, and of the initial formation of thrombin and clot polymerization. During this phase, coagulation factors are assessed as well as the effect of heparin.

- Clot formation time (CFT), from 2 to 20mm: it is the period subsequent to CT, and represents the kinetics of thrombin formation, fibrin polymerization, and clot stabilization by means of involvement of platelets, fibrinogen, and factor XIII.

- Alpha angle: is the angulation described by the patient’s state of coagulability. The more acute it is, the more hypocoagulable; the more obtuse, the greater the tendency towards hypercoagulability.

- Maximum clot firmness (MCF), from 20 to 30mm: period subsequent to CFT, consists in the maximal amplitude of the graphic. Greater stabilization of the clot by fibrin polymerization. It involves interaction among platelets, fibrinogen, and factor XIII. It indicates consistency or quality of the clot, characterizing the patient’s coagulability state.

- A05 to A30: is the firmness of the clot, by the amplitude between the 5 and 30 minute time points.

- Maximum lysis (ML): is the reduction of clot firmness after the MCF. The clot is stable if the ML is less than 15%, or there is hyperfibrinolysis when greater than 15%.

**Figure 3:**
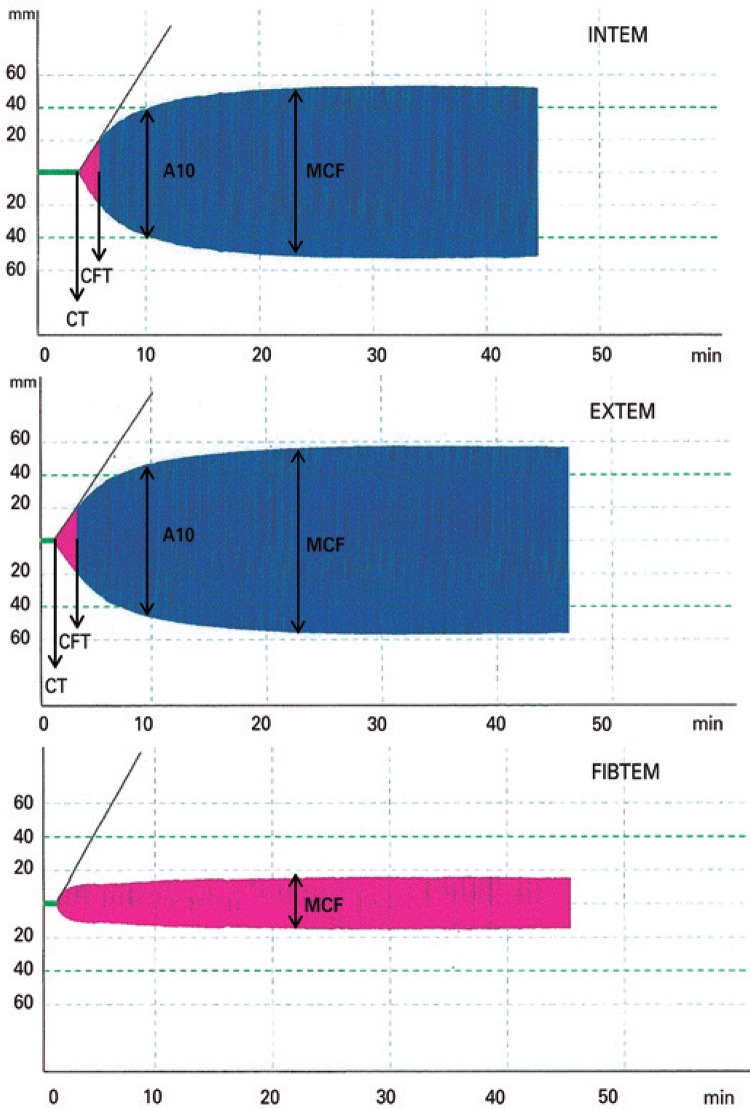
Graphic representation of the different channels (INTEM, EXTEM, and FIBTEM) of rotational thromboelastometry

CT: clotting time; CFT: clot formation time; MCF: maximum clot firmness; A10: amplitude 10 minutes.

The applicability of the variables described is done with the five available tests for real-time analysis of ROTEM^®^: INTEM, EXTEM, FIBTEM, HEPTEM, APTEM (Figures 2 and 3).

- INTEM: activation occurs in the contact phase by ellagic acid. It is sensitive to the intrinsic pathway factors. It evaluates factors XII, XI, IX, VIII, X, V, II, I, and von Willebrand. The CT is more sensitive to non-fractioned heparin >0.15U/mL in the blood.

- EXTEM: activation by thromboplastin or tissue factor (rabbit brain). Onset of clot formation in 70 seconds. It is most sensitive to fibrinolysis. Screening test of the extrinsic pathway: TP (vitamin K-dependent factors: II, VII, IX, X). CT is less sensitive to heparin (>4U/mL of non-fractioned heparin in the blood).

- FIBTEM: activation similar to that of EXTEM. Addition of cytochalasin D inhibits platelet function, allowing isolated activation of fibrinogen. The resulting clot is dependent only on the formation and polymerization of fibrin.

- HEPTEM: activation similar to that of INTEM. Addition of heparinase degraded to heparin is present in the sample. When the HEPTEM corrects the CT alteration, relative to INTEM, this is defined as heparinized blood; otherwise, it represents coagulation factor deficiency.

- APTEM: activation as per EXTEM. By the addition of aprotinin to the reagent, there is inhibition of fibrinolysis. If there is ML correction relative to EXTEM, it characterizes true hyperfibrinolysis (ML>15%).^(2,23)^

## IMPORTANCE OF THROMBOELASTOMETRY

Thromboelastometry provides global information about kinetics and the structure of the clot, besides evaluating the fibrinolytic system.^(24,25)^Viscoelastic tests allow early detection of the coagulation disorder, enabling goal-directed therapy with specific hemostatic agents, according to individual needs (Chart 1).

**Chart 1:** Importance of perioperative monitoring of coagulation by thromboelastometry

Diagnosis of potential causes of hemorrhage
Guide to hemostatic therapies
Risk predictor of bleeding during surgical procedures
Rationalization for blood transfusion
Reducing complications with blood products: TRALI, TACO, TRIM
Monitoring of hypercoagulability states
TRALI: transfusion-related lung injury; TACO: transfusion-associated cardiac overload; TRIM: transfusion-associated immunomodulation.

Source: modified with permission from Lier H, Vorweg M, Hanke A, Görlinger K. Thromboelastometry guided therapy of severe bleeding. Essener Runde algorithm. Hamostaseologie. 2013;33(1):51-61. Review.

The qualitative evaluation of the clot, with graphic representation of the different coagulation disorders, such as hyperfibrinolysis, coagulation factor deficiency, dysfibrinogenemia, platelet dysfunctions, and heparin effect, became a necessity in intensive care unit.^(2,23)^

## CLINICAL APPLICATION

**Figure 4:**
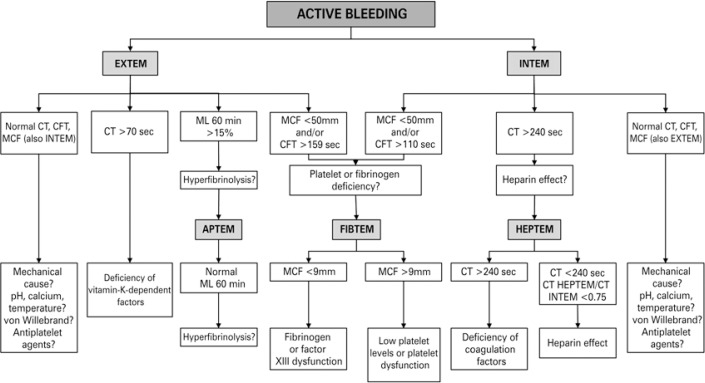
Diagnostic algorithm guided by rotational thromboelastometry

CT: clotting time; CFT: clot formation time; MCF: maximum clot firmness; ML: maximum lysis.

Rotational thromboelastometry screening tests are INTEM and EXTEM. According to preliminary results, we should proceed with the investigation with APTEM and/or FIBTEM and/or HEPTEM, as proposed by Lier et al., (Figure 4).^(2)^

This is an initial screening. In a case of bleeding, one must exclude mechanical causes, hypothermia, acidosis, hypocalcemia, von Willebrand factor deficiency, and prior use of antiplatelet agents. Non-reversal of these etiologies may strive coagulopathy by coagulation factors consumption and impairments in the eletromicrometria profiles. On chart 2, several clinical indications are shown as a benefit of utilizing viscoelastic tests.

**Chart 2:** Indications for thromboelastometry

Coagulopathy associated with trauma and massive hemorrhage
Liver transplant
Cardiac surgery
Major orthopedic surgery and neurosurgery
Hypothermia
Postpartum/neonatal hemorrhage
Hypercoagulability states
Procoagulant therapy
Anticoagulation with non-fractioned heparin

The benefits of goal-directed therapy extend to more rational use of allogeneic blood components, thus reducing their possible iatrogenic effects, such as transfusion-related lung injury (TRALI),^(26)^ transfusion-associated cardiac overload,^(27)^transfusion-associated immunomodulation,^(27)^ venous thromboembolism (VTE), and viral and bacterial infections.^(28,29)^

A multicenter study with 1,175 patients due to blunt trauma and subsequent hemorrhagic shock showed that fresh frozen plasma (FFP) was independently associated with an increase by 2.1% and 2.5% in the incidence of multiple organ failure and acute respiratory distress syndrome, respectively, for each unit transfused.^(30)^ The transfusion of FFP is associated with an increased risk of infection in critically ill surgical patients.^(4)^

Studies demonstrated the cost-effectiveness of ROTEM^®^ due to the reduction of deleterious effects of blood transfusions, costs of blood components, length of hospital stay, and in-hospital mortality.^(20)^

Thromboelastography has limitations that must be considered. The reaction occurs inside the cuvette and not in the endothelium, in a static coagulation mechanism. It is not sensitive to the effect of antiplatelet agents, oral anticoagulants, low molecular weight heparin, and von Willebrand factor deficiency.^(23)^

## CONCLUSION

A new era for thromboelastometry is now. It has changed the paradigm of coagulation analysis in critically ill patients. An early goal-directed therapy is needed for many critically ill patients with coagulopathy. We suggest the routine use of this practical, reproducible and cost-effective tool as the main diagnostic test and as a guide for hemostatic therapy in severe patients with active bleeding.
